# Accuracy in Judging Others’ Personalities: The Role of Emotion Recognition, Emotion Understanding, and Trait Emotional Intelligence

**DOI:** 10.3390/jintelligence8030034

**Published:** 2020-09-18

**Authors:** Cyril Jaksic, Katja Schlegel

**Affiliations:** 1 Department of Psychology, University of Geneva, 1205 Geneva, Switzerland; cyriljaksic@hotmail.com; 2 Institute for Psychology, University of Bern, 3012 Bern, Switzerland

**Keywords:** personality judgments, emotion recognition, emotional intelligence, interpersonal accuracy, impression formation

## Abstract

The ability to accurately judge others’ personality and the ability to accurately recognize others’ emotions are both part of the broader construct of interpersonal accuracy (IPA). However, little research has examined the association between these two IPA domains. Little is also known about the relationship between personality judgment accuracy and other socio-emotional skills and traits. In the present study, 121 participants judged eight traits (Big Five, intelligence, cooperativeness, and empathy) in each of 30 targets who were presented either in a photograph, a muted video, or a video with sound. The videos were 30 second excerpts from negotiations that the targets had engaged in. Participants also completed standard tests of emotion recognition ability, emotion understanding, and trait emotional intelligence. Results showed that personality judgment accuracy, when indexed as trait accuracy and distinctive profile accuracy, positively correlated with emotion recognition ability and was unrelated to emotion understanding and trait emotional intelligence. Female participants were more accurate in judging targets’ personality than men. These results provide support for IPA as a set of correlated domain-specific skills and encourage further research on personality judgment accuracy as a meaningful individual difference variable.

## 1. Introduction

Humans excel at processing sensorial inputs from their environment and infer various kinds of information from them. As social beings, people quickly form first impressions of others’ personality, group membership, and other characteristics, even when only a few cues are available. For example, Willis and Todorov (2006) found that people make relatively stable judgments about strangers’ trustworthiness, competence, or aggressiveness based on pictures that they see for merely 100 ms. People also quickly assess whether an unacquainted person is warm, naïve, kind (Berry and McArthur 1985), successful, popular (Forgas 2011), assertive, cruel, or vulnerable (Berry 1991), and whether a person is liberal or conservative (Olivola et al. 2018).

The “irresistible inclination” to figure out others (Hall et al. 2016a, p. 4) develops early in childhood, with children as young as 3 years old forming similar impressions of trustworthiness, dominance, and competence as compared to adults (Cogsdill et al. 2014). Impression formation can even occur without our awareness (Bargh and Chartrand 1999) and from subliminal exposures (Bargh and Pietromonaco 1982).

Making quick inferences about the personality of others appears to have evolutionary roots and is considered an adaptive process in that it assists us in everyday decisions (Boone and Buck 2003; Haselton and Funder 2006; Zebrowitz and Montepare 2006). Much research has found that first impressions have important real-life consequences. For example, the perceived trustworthiness of defendants affects the evaluation of legal evidence (Porter et al. 2010), the perceived dominance of managers predicts their salary (Graham et al. 2016), and the perceived open-mindedness of men and intelligence of women affects their success in speed-dating (Olivola et al. 2016).

Such findings imply that, in order to be adaptive, first impressions of personality or social characteristics should be accurate. There is accumulating evidence that this is indeed the case—at least to some extent—for traits such as intelligence (Moore et al. 2011), extraversion, conscientiousness, openness (Little and Perrett 2007; Naumann et al. 2009), and narcissism (Shiramizu et al. 2019), and even for characteristics such as sexual orientation, political ideology, or antigay prejudice (Alaei and Rule 2019; Rule et al. 2009; Samochowiec et al. 2010).

The degree to which these variables can be accurately perceived depends both on whether they are expressed in observable cues (e.g., nonverbal behaviors or features of the appearance) in the target individuals and whether the perceivers (judges) detect and use valid cues to make their judgment (Back and Nestler 2016; Funder 2012). Much research in impression formation has focused on verbal and nonverbal cues (e.g., Hirschmüller et al. 2013; Koppensteiner 2013), on comparing different presentation formats (e.g., pictures, videos, face-to-face interactions; Krzyzaniak et al. 2019), and on what makes a “good” (i.e., judgeable) target (Colvin 1993; Funder 1995; Human and Biesanz 2013).

However, comparatively little is known about the “good” (i.e., accurate) judge of personality (e.g., Letzring 2008). In fact, some researchers suggested that the “good judge” might not even exist and individual differences in personality judgment accuracy are small (see Rogers and Biesanz 2019, for a review). To date, there are also no standard tests to measure personality judgment accuracy (Schlegel et al. 2017a). Nevertheless, some studies have identified correlates of the good judge of personality, including self-rated empathic concern and perspective taking (Colman et al. 2017), social skill (Letzring 2008), and communion (Vogt and Colvin 2003). Further, a meta-analysis showed that higher cognitive ability predicted more accurate judgments of personality and other characteristics in human resource management settings (e.g., during job interviews; De Kock et al. 2020).

### 1.1. Personality Judgment Accuracy and Emotion Recognition Ability as Interpersonal Accuracy Domains

The main goal of the present study is to provide further support for personality judgment accuracy as an individual difference variable by demonstrating that it correlates with accuracy in a different domain of interpersonal perception, namely emotion. The ability to accurately recognize emotions in others from nonverbal facial, vocal, and bodily cues (emotion recognition ability, ERA) is, like personality judgment accuracy, part of the broader construct of interpersonal accuracy (IPA; Hall et al. 2016a). IPA refers to the general ability to accurately assess states (e.g., emotions, lying), traits (e.g., personality), and social attributes (e.g., sexual orientation) in others from their verbal and nonverbal behavior (Hall et al. 2016a). Individual differences in ERA have been very widely studied and unlike in the personality judgment domain, several standard tests to measure ERA exist (for an overview, see Bänziger 2016).

Intuitively, one would expect that people who are good at recognizing others’ emotions would also be accurate in judging others’ personalities. Both tasks involve being attuned to the behaviors of others, detecting relevant, often subtle, nonverbal cues, and assigning psychological meaning to their patterns (Boone and Schlegel 2016). Further, it is plausible that basic perceptive abilities such as sensitivity to spatiotemporal forms or the ability to discriminate among subtle variations of auditory and visual cues affect accuracy across all IPA domains (Castro and Boone 2015; Schlegel et al. 2017b). Hall et al. (2016b) also proposed a theoretical model positing that accurately perceiving a target individual’s emotions causally precedes accurate judgments of his or her personality.

However, only a few studies have actually assessed the link between personality judgment accuracy and ERA. In their meta-analysis of correlations between different IPA measures, Schlegel et al. (2017a) identified only three such studies, one of which was unpublished (Hall and Goh 2014) and one of which did not directly report the correlations in the published manuscript (Realo et al. 2003). In both studies, participants judged targets’ personality from short video clips and the accuracy of these ratings was not significantly correlated with standard ERA tests. The third study by Ambady et al. (1995) used a round-robin design in which unacquainted students rated each other’s personalities while sitting at a table (without interacting). This study found a low, but significant positive correlation between personality judgment accuracy and ERA. Another study not included in the meta-analysis examined the association between accurate affect and personality judgments within the same targets and same video material, yielding significant correlations among some emotion–trait pairs but not among others (Hall et al. 2016b). Finally, Schlegel et al. (2020) found small positive correlations among these two IPA domains in older but not younger women. In contrast to the other studies, their design involved a face-to-face interaction between the judge and the (previously unacquainted) target.

One reason for the low and inconsistent associations may be that in most of these studies, personality judgment accuracy was measured using only a few target individuals. As there are important individual differences in targets’ expressivity (Mignault and Human 2019), measuring personality judgment accuracy with only a few targets may not yield reliable estimates of judges’ “true” ability. This might especially be the case if the targets do not vary much in terms of the traits that are being judged. Thus, in order to comprehensively measure judges’ individual differences, a larger number of targets should be included.

### 1.2. Types of Personality Judgment Accuracy

Another important aspect to consider when assessing personality judgment accuracy (and its correlates) is that it can be calculated in different ways using the same data (e.g., Back and Nestler 2016; Hall et al. 2018; Letzring and Funder 2018). The first accuracy type is *trait accuracy*, which refers to the ability to discriminate among different targets on one given trait (e.g., to evaluate whether person A is more or less agreeable than person B), thus requiring inter-target comparisons (Hall et al. 2018). Trait accuracy is typically calculated by correlating judges’ ratings across targets on a given trait with the targets’ self-rated score on this trait (e.g., Lippa and Dietz 2000). This approach was used in the studies of (Hall and Goh 2014; Realo et al. 2003; Hall et al. 2016b), all of which computed single accuracy scores for each of the Big Five traits. Although trait accuracy can be computed for each single trait, one disadvantage is that these single scores are relatively unstable when only a few targets (e.g., six in Hall and Goh 2014; and three in Realo et al. 2003) are judged (Letzring and Funder 2018).

The second accuracy type is *profile accuracy*, which refers to the ability to discriminate relative levels of different traits within one target (e.g., to judge whether person A is more agreeable than extraverted) and thus involves intra-target comparisons (Hall et al. 2018). Profile accuracy is typically calculated by correlating a judge’s ratings for one target across all traits with the target’s self-rated scores on these traits; the correlations for each target can be averaged to form an *overall profile accuracy* score for each judge (e.g., Back and Nestler 2016). This was performed in the studies of Ambady et al. (1995) and Schlegel et al. (2020) in which judges rated targets on 15 and 10 adjectives (traits), respectively.

Because personality profiles of different individuals tend to display similarities (e.g., most people rate themselves as quite agreeable), it is possible to achieve high overall profile accuracy simply by attributing a “normative” (typical) personality profile to each target without necessarily knowing a target’s unique or distinctive constellation of traits (Furr 2008). Another index labeled *distinctive profile accuracy* statistically removes normativeness from overall profile accuracy and yields a measure of a judge’s ability to assess targets’ unique, non-normative personality profiles (e.g., to judge how much person A differs from the typical person on agreeableness and extraversion; Biesanz and Human 2010; Furr 2008). This yields a crucial piece of information because simply attributing the typical profile to each target would inflate a judge’s accuracy score. However, distinctive profile accuracy has been computed in only one study on ERA and personality judgment accuracy (Schlegel et al. 2020).

As shown by Hall et al. (2018), trait accuracy, overall profile accuracy, and distinctive profile accuracy are not interchangeable measures. For instance, single-trait accuracies show only low correlations with each other and with other accuracy types (Hall et al. 2018). These authors also suggested that different mechanisms and cognitive demands may be involved in different accuracy types. Achieving high trait and distinctive accuracy both require the ability to accurately compare targets with each other (on single traits for trait accuracy, or by comparing targets to a “typical” person for distinctive accuracy; Hall et al. 2018). In contrast, high overall profile accuracy requires the ability to accurately rank order traits within a target, that is, making intra-target rather than inter-target comparisons. Hall et al. (2018) proposed that an analytical or local cognitive processing style in which single features or cues in a scene are analyzed selectively may relate more closely to trait and distinctive accuracy, whereas overall profile accuracy may draw more on a global processing style in which all elements of a person’s behavior are analyzed simultaneously. Furthermore, unlike trait and distinctive accuracy, high overall profile accuracy requires (perhaps implicit) knowledge on the personality profile of the average person (Furr 2008).

In order to grasp the full picture of the relationship between the ability to accurately judge others’ personalities and ERA, it is therefore important to analyze all three accuracy types.

### 1.3. The Present Study

The main aim of the present study was to demonstrate that personality judgment accuracy and ERA are positively correlated, as they both represent theoretical facets of the broader construct of IPA (Schlegel et al. 2017a). This was performed by using a more comprehensive measure of personality judgment accuracy than the few existing studies (Ambady et al. 1995; Hall and Goh 2014; Realo et al. 2003; Schlegel et al. 2020).

First, the present personality judgment task used 30 targets (as opposed to one to six targets in the above studies), each of which was judged on eight traits (Big Five traits, intelligence, empathy, and cooperativeness). The Big Five and intelligence have often been studied in impression formation (e.g., Hall et al. 2018; Zebrowitz et al. 2002). Empathy and cooperativeness were added because we believed that these traits were relevant to the type of interaction that the targets had been engaging in (a negotiation roleplay; see method section for details). Second, targets were preselected from a large pool of 130 individuals to include high and low scorers on each of the traits in order to increase variability. Third, targets were shown in one of three modalities (10 targets in each modality): a picture with a neutral facial expression, a 30 s video clip without sound, or a 30 s video clip with sound. Given that IPA appears to involve modality-specific facets (Schlegel et al. 2017a), we included three modalities to measure personality judgment accuracy in a broader fashion.

Based on the personality judgment task including 30 targets, eight traits, and three stimulus modalities, we calculated three total scores for each participant (judge)—trait accuracy, overall profile accuracy, and distinctive profile accuracy. These scores were then correlated with the judges’ performance on a standard ERA test in which participants recognize the emotion displayed by actors in brief video clips with sound (Geneva Emotion Recognition Test, GERT; Schlegel et al. 2014).

We expected that particularly trait and distinctive profile accuracy would positively correlate with ERA, as all three variables seem to benefit from analytical or local cognitive processing of the behavioral cues of targets (Hall et al. 2018; Martin et al. 2012). Overall profile accuracy was expected to show lower associations with ERA, because the latter does not entail knowledge on typical personality profiles of people and does not require making intra-target comparisons. We also planned to conduct additional analyses for accuracy subscores by trait and by modality. Although these analyses were exploratory, we expected that ERA would be more highly correlated with personality judgment accuracy when targets were shown in video clips rather than as still pictures, because videos match the GERT stimulus material more closely.

The second aim of the present study was to examine the discriminant validity of personality judgment accuracy with respect to other socio-emotional variables, namely emotion understanding and trait emotional intelligence, as well as with gender. Emotion understanding can be defined as knowledge about the causes, characteristics, and consequences of emotions and is typically measured with performance-based tests that have correct and incorrect responses (MacCann and Roberts 2008). Emotion understanding is substantially correlated with ERA and both constructs are part of the broader concept of ability emotional intelligence (Schlegel and Scherer 2018). It might be that emotional understanding relates to better knowledge about people’s typical personalities and about which cues signal different traits, and therefore correlates with making accurate personality judgements. On the other hand, emotion understanding is typically measured with text-based scenarios (e.g., MacCann and Roberts 2008) and might not tap into more accurate processing of non-emotional verbal and nonverbal cues. Given that no previous study assessed the link between personality judgment accuracy and emotion understanding, we did not specify a hypothesis and analyzed this association exploratively.

Trait emotional intelligence (trait EI) refers to a set of affect-related traits, motivations, and other dispositions that are measured with self-report questionnaires (Petrides and Furnham 2003). Depending on the specific theoretical model used, trait EI incorporates components such as emotional self-awareness, stress tolerance, self-control, emotional well-being, or sociability (Petrides 2011). On the one hand, some previous studies found personality judgment accuracy to correlate with similar traits such as empathic concern and perspective taking (Colman et al. 2017), communion (Vogt and Colvin 2003), or social skill (Letzring 2008). On the other hand, self-reported EI and related socio-affective traits typically show no or only small correlations with ERA or other emotional abilities as measured with performance-based tests (Hall et al. 2009; Murphy and Lilienfeld 2019; Riggio and Riggio 2001; Roberts et al. 2010).

As trait EI has previously not been assessed as a correlate of personality judgment accuracy, we analyzed this relationship in an exploratory fashion.

With respect to gender, a small but significant advantage for women has been well established in the literature on ERA (e.g., Hall 1978; Thompson and Voyer 2014). For personality judgment accuracy, results are more mixed, with some studies finding that women performed better than men (Ambady et al. 1995; Letzring 2008, Study 2; Vogt and Colvin 2003; average effect size across these three studies *r* = .25) and other studies finding no significant differences with correlation coefficients close to zero (Christiansen et al. 2005; Letzring 2008, Study 1; Lippa and Dietz 2000). Here, we hypothesize that because both domains are part of the broader skill of IPA, women will be more accurate in judging targets’ personality traits than men.

Taken together, the goal of the present study was to establish personality judgment accuracy more firmly as a facet of IPA by demonstrating positive associations with ERA and a higher performance of female judges. Emotion understanding and trait EI were analyzed as correlates in an exploratory fashion.

## 2. Materials and Methods

### 2.1. Stimulus Creation

The targets were selected from among 130 students (graduate and undergraduate) and staff members who took part in a study conducted at the University of Geneva (51% male, 49% female; age M = 23.5 years, SD = 4.0 years). They were filmed by a medium shot camera and their speech was recorded with a clip-on microphone during a negotiation task with another participant (see Schlegel et al. 2018, for further details). After the negotiation, participants were also asked to pose with a neutral expression for a front-facing headshot with a black background. In both filming and photographing, participants wore uniform black clothes, their hair style was kept to minimal eccentricity and flashy jewelry was removed.

Prior to the negotiation, participants completed the Big Five Inventory (BFI; Plaisant et al. 2010), the Interpersonal Reactivity Index (IRI, Davis 1983), and the NV5-R general reasoning subtest (Thiébaut and Bidan-Fortier 2003).

The BFI is a 45-item self-report questionnaire yielding scores for openness for experience, conscientiousness, extraversion, agreeableness, and neuroticism. The IRI is a 28-item self-report questionnaire assessing multiple facets of empathy including empathic concern, fantasy, perspective taking, and personal distress. As described in more detail below, the empathic concern score was used as the criterion for assessing accuracy in judging targets’ empathy because this scale corresponded best to the meaning we assumed laypeople would attribute to the term empathy (being caring, sensitive, and understanding). The NV5-R is a 20-min performance-based test measuring inductive and deductive reasoning with numeric, spatial, and lexical content which yields one total intelligence score and has been validated with French speakers. After the negotiation, participants rated their own cooperativeness during the interaction using nine items such as “Throughout the negotiation, I tried to be cooperative.” (for the complete list of items, see Schlegel et al. 2018; Cronbach’s alpha = .80). These items yielded one total cooperativeness score. The values on all measures were standardized to be on the same scale ranging from 1 to 5.

Among the 130 participants of the negotiation study, 15 male and 15 female targets were chosen so that there would be at least one target scoring high and one scoring low on each of the eight following dimensions, corresponding to the constructs assessed with the instruments described above: openness to experience, conscientiousness, extraversion, agreeableness, neuroticism, cooperativeness, empathy, and intelligence. Participants were not eligible to be selected as targets if technical problems had occurred during the recordings, if they were acquainted with their negotiation partner, or if they wore visible piercings.

The 30 selected target individuals were then randomly distributed to the three exposure modalities: picture, mute video, and audio video, while ensuring that for each modality, targets were allocated equally in terms of gender and personality dimensions that they had high or low scores in. The final stimuli were five males and five females for each of the three exposure modalities. The pictures with a neutral expression (taken after the negotiation) were used as stimuli for the picture modality, and the videos of the first 30 s of the negotiation were used for both the audio video modality and the muted video modality. Targets’ scores on the eight dimensions and their allocation to the three exposure modalities can be seen in Appendix A.

### 2.2. Participants and Procedure

Participants (judges) were recruited through flyers and advertisements at the University of Geneva, Switzerland. A total of 121 participants (61% female, 39% female), with a mean age of 23.6 (SD = 4.7) years, took part in the present study.

Participants completed this study in small groups in the same room, each of them sitting in front of a computer with headphones on. This study was administered in French. Participants were first orally briefed about the procedure and the upcoming tasks. On the computer, instructions explained the first task—reporting one’s first impression of different targets on eight dimensions: open to new experiences, conscientious, extraverted, agreeable, moody (considered as more accessible than *neurotic*), empathic, cooperative, and intelligent. A brief definition was provided for some dimensions to ensure all participants had the same understanding of the labels (see Appendix B for the full instructions).

After seeing the picture or video of a target, participants had to rate each of the eight adjectives on a 5-point Likert scale. The instructions specified that if the participant knew a target individual, they were still required to assess them but had to tick a box at the bottom of the page. The ratings for acquainted targets were excluded from the analyses (this was the case for 34 out of the 3630 ratings made by all participants; no participant knew more than 5 out of the 30 target individuals). After one practice trial, the 30 targets were presented in a way that the exposure modalities were semi-randomized so that the same modality would not appear more than four times in a row. Once the 240 evaluations (30 targets * 8 traits) were made, participants completed measures of ERA, Emotion Understanding, and trait EI (see next section), and provided basic demographic information. The whole session lasted about 50 min and participants were paid 15 CHF for their participation. This research was approved by the ethics committee of the University of Geneva (no code assigned).

### 2.3. Measures

#### 2.3.1. Emotion Recognition Ability: GERT

The Geneva Emotion Recognition Test (GERT) (Schlegel et al. 2014) was used to measure the judges’ ERA. This test includes 83 short videos of actors displaying 14 different emotions (pride, joy, amusement, pleasure, relief, interest, anxiety, fear, despair, sadness, disgust, irritation, anger, and surprise) while pronouncing a meaningless sentence in order to convey emotional prosody. The videos show the actors’ upper body and faces. Participants have to infer the emotion being displayed in each clip by choosing the correct term out of 14 presented after the video. A total ERA score is calculated as the percentage of correctly recognized clips. The GERT has demonstrated good reliability and validity in previous studies (Schlegel et al. 2014; Schlegel et al. 2019) and captures the emotion perception component in (Mayer and Salovey 1997) ability EI model. Cronbach’s alpha was .89.

#### 2.3.2. Emotion Understanding: STEU

The Situational Test of Emotion Understanding (STEU) (MacCann and Roberts 2008) is a widely used performance-based test and consists of 25 items that assess one’s ability to infer emotional states from written scenarios. Different situations are described and, for each of them, participants have to choose the emotion the character is most likely feeling. Some items feature the opposite task; the feeling of a character is described, and participants have to identify the event that most likely induced such a feeling. This test captures the emotion understanding/emotion knowledge component of (Mayer and Salovey 1997) ability EI model. Cronbach’s alpha was .78.

#### 2.3.3. Trait Emotional Intelligence: TEIQue-SF

The Trait Emotional Intelligence Questionnaire short form (TEIQue-SF) (Petrides and Furnham 2006) is a widely used questionnaire which asks participants to rate, on a 7-point Likert scale, the extent to which 30 emotion- and mood-related statements apply to them (e.g., “I usually find it difficult to regulate my emotions”). The TEIQue-SF captures four facets of trait EI (well-being, self-control, emotionality, sociability) which are combined into one total score. Cronbach’s alpha was .93.

#### 2.3.4. Personality Judgment Accuracy

Three accuracy scores (trait accuracy, overall profile accuracy, and distinctive profile accuracy) were computed using targets’ BFI scores (for openness to experience, conscientiousness, extraversion, agreeableness, and neuroticism), IRI empathic concern scores, self-reported cooperativeness, and NV5-R scores (for intelligence) as criteria. All of the criterion scores had been standardized to range from 1 to 5 to correspond to the rating scale used by the judges.

In order to calculate *trait accuracy*, for each of the trait by modality combinations (8 traits × 3 modalities = 24 combinations), each participant’s 10 ratings (one per target) were correlated with the 10 “true” values (one per target). For example, accuracy in judging agreeableness in the picture modality was calculated for each participant (judge) by correlating his/her 10 ratings with the self-reported agreeableness scores of each target. If all 10 targets in one modality were rated with the same value, no correlation could be computed, and a missing value was assigned to this modality/trait combination. The resulting 24 correlations were Fisher z transformed and then averaged to form one total trait accuracy score (only if at least 20 correlations, i.e., >80%, were available). This procedure corresponds to previous studies (e.g., Hall et al. 2018).

Higher trait accuracy scores imply a better ability to discriminate targets on a given trait (e.g., to evaluate whether target A is more or less extraverted than target B). For exploratory purposes, we also calculated subscores for the three modalities (by averaging the scores of all eight traits within each modality) and for the eight traits (by averaging the scores of all three modalities within each trait).

Overall and distinctive profile accuracy were calculated following the procedure described by (Furr 2008). In order to calculate *overall profile accuracy*, for each of the 30 targets, the 8 ratings (one on each trait) provided by a judge were correlated with the 8 “true” values for the target. The resulting 30 correlations per judge were Fisher z transformed and averaged to form one total overall profile accuracy score for each judge (only if at least 24 correlations, i.e., 80% or more, were available). Higher scores imply a better ability to distinguish the relative levels of different traits within targets (e.g., to evaluate whether target A is more extraverted than agreeable).

*Distinctive profile accuracy* was calculated as follows: first, the mean rating on each trait provided by all judges across all targets was subtracted from each target’s individual rating provided by a judge, yielding a distinctive rating profile for each target. Second, the mean self-rating or objective test score (for intelligence) on each trait across all 30 targets was subtracted from each target’s individual self-rating or test score, yielding a distinctive criterion profile for each target. For each of the 30 targets and each judge, the distinctive rating profile (consisting of 8 values, one per trait) was correlated with the distinctive criterion profile. The resulting 30 correlations per judge were Fisher z transformed and averaged to form one total distinctive profile accuracy score (for participants with 24 or more available correlations). Higher values imply a better ability to understand how a target’s trait profile differs from that of other people and from the average person (e.g., to evaluate whether target A is relatively more extraverted and less agreeable than the average person).

For exploratory purposes, we also calculated overall and distinctive profile accuracy scores for the three modalities by averaging the respective profile correlations for the 10 targets within each modality (picture, mute video, audio video).

Reliability was .36 for trait accuracy (Cronbach’s alpha over the 24 individual scores), .17 for overall profile accuracy, and .16 for distinctive profile accuracy (calculated as “replicability” indices using the R package *multicon* for the analysis of profile correlations; Sherman 2015). Although these values are low compared to traditional psychometric standards, they are in the range of what other researchers have reported for similar tasks (e.g., Rogers et al. 2018; Schlegel et al. 2020) and they are in line with the generally low reliability found for IPA tasks (Schlegel et al. 2017a). It should also be noted that the present study used 30 targets, which is substantially more than other studies which often use fewer than 10 targets (e.g., Hall et al. 2018), and included eight traits, which meets the minimum recommendation for profile accuracy studies by (Rogers et al. 2018).

## 3. Results

### Descriptive Statistics

Descriptive statistics for all main measures and their intercorrelations can be found in Table 1. As GERT, STEU, TeiQue, and distinctive profile accuracy were not normally distributed, Spearman rank correlations were used. Descriptive statistics and correlations with GERT, STEU, TeiQue, and gender for the personality judgment subscores by trait and modality are presented in Table 2. Zero-order correlations among all accuracy subscores and total scores are provided in Appendix C.

One-sample t tests showed that all three personality judgment accuracy scores were significantly bigger than zero (trait accuracy, t(114) = 6.443, *p* < .001; overall profile accuracy, t(115) = 11.757, *p* < .001; distinctive profile accuracy, t(118) = 7.440, *p* < .001). The three scores showed substantial positive intercorrelations, with distinctive profile accuracy and trait accuracy showing the highest association (*r* = .72, *p* < .001) in line with (Hall et al. 2018).

With respect to our hypotheses, as expected, results showed that GERT scores were positively and significantly correlated with trait accuracy and with distinctive profile accuracy, but not with overall profile accuracy. Effect sizes were small to moderate. When correcting these correlations for measurement error due to the imperfect reliability of GERT and accuracy scores (Schmidt and Hunter 2004), the effect sizes increased in magnitude (GERT and trait accuracy, *r* = .52; GERT and overall profile accuracy, *r* = .28; GERT and distinctive accuracy, *r* = .51). STEU and TeiQue were largely unrelated to all three personality judgment accuracy scores (all correlations below *r* = .10).

Female participants achieved significantly higher trait accuracy and profile accuracy scores than men. Additionally, we examined whether accuracies were higher when judges rated targets of the same gender (i.e., men rating men and women rating women). Trait accuracies split by target gender were as follows: men rating male targets M = .00 (SD = .11), men rating female targets M = .03 (SD = .12), women rating male targets M = .03 (SD = .12), and women rating female targets M = .07 (SD = .11). Overall profile accuracies were as follows: men rating male targets M = .06 (SD = .12), men rating female targets M = .09 (SD = .14), women rating male targets M = .11 (SD = .12), and women rating female targets M = .13 (SD = .10). Finally, distinctive profile accuracies were as follows: men rating male targets M = .04 (SD = .11), men rating female targets M = .04 (SD = .10), women rating male targets M = .04 (SD = .10), and women rating female targets M = .06 (SD = .09). For trait accuracy and overall profile accuracy, male–male ratings were significantly less accurate than female–female ratings (t(118) = −3.40, *p* < .001; and t(119) = −3.47, *p* < .001, respectively). For distinctive profile accuracy, male–male ratings and female–female ratings did not differ significantly (t(119) = −1.09, *p* = .277), but they had almost the same values as cross-gender accuracy scores. Taken together, these results speak against an own-gender effect in personality judgment accuracy.

Table 2 shows that participants were able to judge all traits significantly better than chance, except for conscientiousness, neuroticism, and empathy. These results confirm past research showing that neuroticism and conscientiousness are not among the most accurately judged traits (e.g., Back and Nestler 2016; Naumann et al. 2009); for empathy, to our knowledge, no other studies are available. Comparing the three modalities, accuracy was the lowest in the photo modality and not significantly above chance when trait accuracy in the photo modality was computed. However, profile accuracy scores were higher than chance in the photo modality as well.

Exploratory analyses showed that GERT scores correlated positively with trait accuracy in all traits, although only the correlations for intelligence and neuroticism reached significance. GERT scores were significantly correlated with accuracy in judging others’ traits in mute videos, but not in pictures or in videos with sound. For distinct and overall profile accuracy, none of the modalities reached a significant correlation with the GERT.

The STEU and TeiQue were unrelated to personality judgment accuracy subscores. The additional analyses also revealed that the female advantage in accurately judging personality was most pronounced in the muted video domain.

## 4. Discussion

While research on the accuracy of first impressions of others’ personality has had a long tradition, studies on individual differences in personality judgment accuracy and its correlates have been relatively rare (e.g., Letzring 2008). In particular, although the ability to make accurate personality judgments has been proposed as an integral facet of IPA, little is known about how it relates to another important facet, namely the ability to accurately judge others’ emotions (ERA). Furthermore, to our knowledge, no study to date has assessed whether personality judgment accuracy relates to emotion understanding—a performance-based component of ability EI—and self-reported trait EI.

The present study showed that, in line with our expectations, two indicators of personality judgment accuracy were significantly correlated with ERA: trait accuracy, which reflects the ability to accurately rank targets on each personality trait, and distinctive profile accuracy, which reflects the ability to judge how much a target’s trait levels deviate from the average person. Raw effect sizes were small to medium, but when applying an attenuation correction, they were large (around *r* = .50). Overall profile accuracy, which is the ability to judge targets’ personality profiles without partialling out the personality profile of an average person, showed only a small, nonsignificant positive correlation with ERA (and was small to medium when corrected for attenuation). Emotion understanding and trait EI were unrelated to accuracy in judging personality.

These results are in line with the assumption that ERA, trait accuracy, and distinctive accuracy may share common mechanisms and underlying skills such as a higher sensitivity to low-level auditory and visual signals and a higher ability to detect and interpret relevant (often subtle and brief) nonverbal cues (Castro and Boone 2015). Performance on all three variables may also benefit from a more analytical, local processing style of these cues as suggested by Hall et al. (2018). It can also be speculated that these scores are more strongly based on making distinctions between targets and other people (e.g., by comparing an individual target’s cues to prototypical schemas), whereas a high overall profile accuracy score can be achieved by simply attributing a typical profile to all targets without making fine-grained individual distinctions. While this type of accuracy appears to be relatively distinct from ERA, it might still be a valid indicator of how well a person can grasp the personality profile of a new acquaintance, a job candidate, etc., in everyday life (Hall et al. 2018; Letzring and Funder 2018).

These findings have several implications for research on IPA and personality judgements. First, they highlight that different ways of calculating personality judgment accuracy do not yield interchangeable results and that each accuracy type may reflect somewhat different psychological mechanisms (e.g., making inter- vs. intra-target comparisons; Hall et al. 2018). Future studies should therefore ideally calculate all accuracy types, although this requires a judgment task with many targets and many traits (Letzring and Funder 2018).

Second, these results help establish personality judgment accuracy more firmly among the central IPA domains. Whereas in the meta-analysis of Schlegel et al. (2017a), the average correlation between the personality and the emotion domains was only *r* = .09, the effect sizes found here were substantial for both trait and distinctive profile accuracy. The present results are thus more in line with the theoretical conceptualization of IPA as a broad skill that subsumes all domains of person perception (Hall et al. 2016a) and suggest that people who are accurate in detecting and interpreting others’ states (emotions) are also better at judging others’ more stable traits. This interpretation is further supported by the female advantage that emerged in both the personality and emotion judgment tasks (Table 1), which corresponds to well-established findings in the ERA domain (Thompson and Voyer 2014). Taking a closer look at the different modalities, however, the correlations with gender were close to zero for the audio-visual condition, which provided the richest information for judging personality (speech in addition to visual nonverbal cues). Given that overall accuracy was higher in the audio video than in the mute video condition, it might be that men benefit particularly from verbal content and/or nonverbal auditory cues (prosody) when judging personality. At least in the ERA field and for nonverbal cues (i.e., stimuli without verbal content), however, the gender difference seems to be of similar magnitude across the visual and auditory modalities (Thompson and Voyer 2014). In Hall’s (1978) meta-analysis, the female advantage in affect perception was actually larger for the combination of visual and auditory stimuli than for single modalities. In the domain of personality judgment accuracy, to our knowledge, no other study has compared gender differences across different modalities.

There are several possible explanations for why the correlation among the personality and emotion domains of IPA is higher in the present study than in previous research. One reason may be that the present study captured personality judgment accuracy in a broader way than previous studies, in particular by including a large number of targets that were preselected to cover high and low scorers on the assessed traits, increasing variation. This may have yielded a more internally valid and stable estimate of participants’ skill (Bollen and Lennox 1991; Letzring and Funder 2018). Another likely reason is that in two out of the three studies in the existing meta-analysis (Schlegel et al. 2017a), single-trait accuracy scores (e.g., accuracy in judging extraversion) rather than an aggregated trait accuracy score had been correlated with ERA (Hall and Goh 2014; Realo et al. 2003; see also Hall et al. 2016b). As Schlegel et al. (2017a) noted, IPA appears to be a set of only loosely related domain-, modality-, and trait-specific skills or facets, and measuring many of these relatively unique facets will lead to a more valid estimate of a person’s global IPA (see also Bollen and Lennox 1991, for the psychometric underpinnings of this model). Applying this logic to the personality domain, aggregating accuracies across single traits (which typically show very low intercorrelations; e.g., Hall et al. 2018) should provide a more internally valid and stable estimate of personality judgment accuracy. This can be seen in the present data, where the average correlation of the eight single-trait accuracies with ERA (see Table 2) is only *r* = .11, while the correlation with the aggregate score is *r* = .33.

A third implication of this study is that it is in line with the State and Trait Accuracy Model (STAM) which proposes that emotion recognition and personality attributions are part of the same general process of person perception, with perceptions of brief affective states directly preceding and informing judgments about a target’s personality (Hall et al. 2016b). However, the present study was correlational and did not assess emotion and personality judgments in the same targets, which would be necessary to directly test the STAM.

This study is the first, to our knowledge, to assess emotion understanding and trait EI as correlates of personality judgment accuracy. Both measures were not significantly associated with personality judgment accuracy. Thus, being good at evaluating how agreeable, intelligent, or neurotic someone is does not seem to rely on general crystallized knowledge about emotions (MacCann and Roberts 2008) and is also unrelated to self-perceptions of oneself as emotionally attuned, stress resistant, happy, and sociable (Petrides and Furnham 2003). However, other studies have provided some evidence that traits signaling a high interpersonal orientation such as empathic concern go along with more accurate personality judgments (e.g., Colman et al. 2017). More research is therefore needed to further examine the nomological network of accurate personality judgments, both within and outside of IPA (De Kock et al. 2020).

Within the IPA domain, for instance, it would be useful to assess both ERA and personality judgments in separate modalities (e.g., audio only, picture only, video only) in order to better understand the modality-specific contributions to both IPA domains. Future studies should also examine the relationship between ERA and accurate personality judgments when judges and targets are acquainted. Outside the IPA domain, examining the association with personal intelligence—the ability to reason about personality and personality-relevant information—would be a logical step (Mayer et al. 2012).

Future studies looking into the correlates and the predictive validity of individual differences in personality judgment accuracy would highly benefit from the creation of a standard test to assess these. Such a test would also allow comparing the overall accuracy levels of people in the personality and emotion judgment domains when stimuli are preselected by using the *pi* metric proposed by (Hall et al. 2008).

However, such an endeavor is highly complex. Below, we briefly address some of the decisions and considerations to be made when developing such a test and discuss how the present study informs these.

First, a decision has to be made about the type of stimulus material and the modality of stimulus presentation. In the present study, accuracy was quite low in the picture modality and was not significantly correlated with ERA, whereas the two video modalities showed higher accuracies and higher correlations. This suggests that individual differences in personality judgment accuracy may be better captured when using video clips. Alternatively, the correlations may have been higher in the video modalities because the GERT is also based on videos and not pictures, and a picture-based ERA test may have yielded higher associations with accurate personality judgments from photos. Nevertheless, a standard test using videos rather than pictures would also ensure a higher ecological validity, as impression formation in everyday life is often based on multimodal and dynamic information.

Second, if video clips are used, an appropriate setting in which the targets are presented must be chosen. Previous studies have used a variety of settings, including reading a standard text (Borkenau and Liebler 1993), playing a word-guessing game (Human and Mendes 2018), engaging in a getting-acquainted conversation (Hall et al. 2016b) or in a mock job interview (Krzyzaniak et al. 2019). It is plausible that some settings provide better opportunities to observe cues relevant for a given trait than others (Funder 2012), but little is known about which scenarios and which traits show the best “fit” in terms of judgeability. Ideally, a standard test would display targets in a way that ensures that cues relevant to each of the judged traits are present. That is, the development of a new test would require matching each included trait with a suitable setting.

The setting used in the present study (beginning of a negotiation roleplay with an unacquainted person) appeared to be useful for judging most traits, with extraversion, intelligence, openness, agreeableness, and cooperativeness being judged better than chance. On the other hand, accuracy in judging neuroticism (which was not judged better than chance) was nevertheless significantly correlated with ERA (see Table 2). This implies that there may be meaningful individual differences in accuracy for traits that are, on average, difficult to judge. However, some “easier” traits (e.g., extraversion) did not significantly correlate with ERA. An additional possible consideration is that for some traits, targets’ self-ratings may be biased by socially desirable responding. The development of a standard accuracy test therefore requires a careful validation of each included trait.

A third consideration is the selection of targets for the test. It is known that some targets are more judgeable than others (Colvin 1993; Mignault and Human 2019). Targets that have low expressive accuracy and are hard to be judged would, in psychometric terms, result in very difficult (or unsolvable) test items because the stimuli would contain very few or no observable cues to the traits in question. The fact that targets are typically not preselected with respect to their judgeability in studies on personality judgments may contribute to the low internal consistency usually observed for accuracy scores (Rogers and Biesanz 2019). Low reliability estimates were also found in the present study which preselected targets on their self-rated (and for intelligence, objectively measured) trait levels but not regarding whether they represent a “good target”. From a psychometric point of view, targets that are not at all judgeable (i.e., representing “unsolvable items”), should thus not be part of a standard test. On the other hand, including only “good targets” might make the test too easy and might reduce the variability of the target pool on characteristics such as adjustment, personality coherence, and social skills that are higher in judgeable people (Mignault and Human 2019). Thus, a test should ideally include targets with differing levels of judgeability (“item difficulty”) above a certain minimal threshold.

A final consideration concerns cultural and language-related aspects. In the present study, adding audio did not substantially increase accuracy compared to the muted video condition, and for trait accuracy, the muted video modality yielded the highest correlation with ERA. It might thus be appropriate to develop a standard test using only the video channel, which would have the advantage that it could be used in multiple languages. On the other hand, it is likely that in complex and naturalistic settings, targets will display culture-specific nonverbal dialects, which might require developing separate tests for different cultures nevertheless (Elfenbein et al. 2007).

Taken together, the present results add to our limited understanding of individual differences in personality judgment accuracy and provide empirical support for the broader concept of IPA. However, much more research as well as standard tests are needed to further examine the nomological network as well as the predictive validity of personality judgment accuracy for outcomes such as relationship quality, professional success, or well-being. Such endeavors might highly benefit from the vast literature in its sister IPA domain, emotion recognition (for an overview, see Schmid Mast and Hall 2018).

## Figures and Tables

**Table 1:** Descriptive Statistics and Intercorrelations (Spearman Rank Correlations) of Main Study Variables.

	Mean (SD)	N	(1)	(2)	(3)	(4)	(5)	(6)
(1) GERT	65.43 (11.19)	120						
(2) STEU	15.72 (3.50)	121	.40 ***					
(3) TEIQue	149.65 (19.07)	121	−.08	.01				
(4) trait accuracy	.06 (.09)	115	.33 ***	.08	−.12			
(5) overall profile accuracy	.13 (.12)	116	.11	.04	.09	.56 ***		
(6) distinctive profile accuracy	.06 (.08)	119	.21 *	.03	−.07	.72 ***	.70 ***	
gender (0 = male, 1 = female)	.61	121	.22 *	.10	−.03	.27 **	.25 **	.14

GERT = Geneva Emotion Recognition Test; STEU = Situational Test of Emotional Understanding; TeiQue = Trait Emotional Intelligence Questionnaire; * *p* < .05, ** *p* < .01, and *** *p* < .001.

**Table 2:** Descriptives for Personality Judgment Subscores and Spearman Rank Correlations with GERT, STEU, and TeiQue.

	Mean (SD)	GERT	STEU	TeiQue	Gender (0 = Male, 1 = Female)
trait accuracy subscores					
openness	.08 (.24) ***	.19 *	.08	−.07	.21 *
conscientiousness	.03 (.22)	.08	.01	.14	−.07
neuroticism	−.01 (.23)	.20 *	.11	−.12	.15
agreeableness	.04 (.19) *	.11	.17	.03	.17
extraversion	.15 (.23) ***	.12	−.02	−.15	.01
empathy	.00 (.21)	.11	−.04	−.10	.16
cooperativeness	.08 (.24) ***	.12	.11	.05	.11
intelligence	.07 (.20) ***	.21 *	−.10	−.06	.14
trait accuracy photo	.03 (.13) *	.13	−.12	−.07	.19 *
trait accuracy mute video	.06 (.15) ***	.29 **	.12	−.11	.26 **
trait accuracy audio video	.08 (.14) ***	.18	.08	.00	.04
profile accuracy subscores					
overall accuracy photo	.05 (.18) **	.18	.12	.09	.14
overall accuracy mute video	.19 (.20) ***	.00	−.03	.00	.26 **
overall accuracy audio video	.16 (.17) ***	.05	.02	.09	.06
distinctive accuracy photo	.08 (.16) ***	.14	.04	.01	−.05
distinctive accuracy mute video	.08 (.18) ***	.13	.04	−.10	.26 **
distinctive accuracy audio video	.04 (.14)	.05	−.06	.11	−.06

Asterisks for means refer to *p*-values in one-sample t tests against zero. GERT = Geneva Emotion Recognition Test; STEU = Situational Test of Emotional Understanding; TeiQue = Trait Emotional Intelligence Questionnaire; * *p* < .05, ** *p* < .01, and *** *p* < .001.

## Appendix A

**Table A1:** Demographic and Personality Characteristics of the 30 Targets Used in the Personality Judgment Accuracy Task.

Target ID	Sex	Age	Extra	Conscien	Neuro	Open	Agree	Coop	Intel	Emp	Mod
1	M	34	2.4	2.3	3.0	3.4	3.5	3.1	.6	2.0 ↓	a-v
2	M	33	2.9	3.8	2.9	3.9	4.7	4.4	.8	4.6 ↑	a-v
3	F	21	3.4	3.4	4.4	3.3	4.2	3.4	.9 ↑	3.9	a-v
4	M	18	3.8	2.6	1.5 ↓	4.4	3.8	3.8	.8	3.4	a-v
5	M	33	3.9	4.6	1.8	4.5	4.9 ↑	5.0	.6	3.9	a-v
6	F	26	4.1	2.8	2.9	4.5	3.6	5.0 ↑	.8	4.6	a-v
7	F	22	4.3	5.0 ↑	3.3	3.2	4.0	3.1	.6	3.3	a-v
8	F	21	4.8	4.1	2.6	2.8	3.2	4.0	.6	2.6 ↓	a-v
9	F	22	4.8	2.2 ↓	3.8	2.9	3.9	3.3	.6	4.1	a-v
10	M	20	1.6 ↓	4.3	2.3	3.4	4.0	3.9	.8	3.3	a-v
11	M	21	3.1	3.2	3.9	4.2	2.5 ↓	3.4	.8	3.1	m-v
12	F	21	3.5	4.9	1.1 ↓	4.2	4.2	4.4	.8	4.4	m-v
13	M	20	3.5	3.0	2.4	4.7 ↑	2.4	3.9	.9	1.7 ↓	m-v
14	M	22	3.6	1.9 ↓	2.4	3.4	3.2	2.7	.8	3.7	m-v
15	F	25	4.1	4.3	1.0 ↓	4.0	4.9 ↑	5.0	.4	3.9	m-v
16	F	24	4.1	3.2	1.8	3.5	4.3	4.4	.4	4.9 ↑	m-v
17	F	28	4.3	4.0	1.9	3.6	4.5	3.9	.4 ↓	4.1	m-v
18	M	19	4.8	3.6	3.9 ↑	4.4	4.0	3.9	.8	3.9	m-v
19	F	22	1.6 ↓	3.8	4.3	3.8	4.3	4.7	.7	4.3	m-v
20	M	30	4.9 ↑	4.1	2.3	3.6	4.2	4.8	.8	3.7	m-v
21	F	23	2.5	4.3	3.8	4.7 ↑	4.2	4.6	.7	4.3	pic
22	M	24	2.6	2.8	2.6	3.8	3.8	4.3	.9 ↑	3.7	pic
23	M	31	2.6	4.3 ↑	1.6	3.1	3.8	4.5	.4	4.7	pic
24	F	23	3.0	3.2	3.5	4.0	2.9 ↓	3.6	.5	3.1	pic
25	M	22	3.1	3.9	3.6	3.9	3.7	4.9 ↑	.6	4.1	pic
26	M	32	3.6	4.2	2.4	3.6	4.2	4.0	.2 ↓	3.7	pic
27	F	24	4.0	3.7	4.6 ↑	4.6	4.0	2.3	.6	4.7	pic
28	M	20	4.0	3.9	2.0	2.3 ↓	4.3	4.1	.6	4.6	pic
29	F	21	4.6	3.0	3.0	2.6 ↓	3.8	3.2	.5	4.4	pic
30	F	23	5.0 ↑	3.8	2.6	4.6	4.4	3.8	.5	4.0	pic

Extra = extraversion, conscien = conscientiousness, neuro = neuroticism, open = openness to experience, agree = agreeableness, coop = cooperativeness, intel = intelligence, emp = empathy, mod = modality, a-v = audio video, m-v = mute video, and pic = picture. The arrows reflect the high (↑) or low (↓) score for which the target has been selected.

## Appendix B

Instructions Provided to Participants for Personality Judgments

You will be presented pictures and videos with or without sound featuring people simulating a job interview. Your task is to provide your first impression of these people, as you imagine them to be in real life. There is no right or wrong answer. We are interested in impression formation, as subjective as it is.

If you know a person, please evaluate him or her anyway. Click then on the “yes” button at the bottom of the slide to indicate that this person is familiar to you. Once the evaluation is over, simply click on “next…” for the next evaluation. You will start with a practice trial (a picture presented for 8 s) so you can get familiar with the task.

Terms and descriptions provided for the evaluations:

**Open to new experiences**: creative, gets interested in many things
**Conscientious**: organized, persevering, efficient
**Extraverted**: sociable, expressive, confident
**Agreeable**: helpful, thoughtful, lenient
**Moody**: easily distressed, easily tormented
**Empathic**: sensitive, understanding
**Cooperative**
**Intelligent**

## Appendix C

**Table A2:** Spearman Rank Correlations among All Accuracy Measures and Subscales.

	(1)	(2)	(3)	(4)	(5)	(6)	(7)	(8)	(9)	(10)	(11)	(12)	(13)	(14)	(15)	(16)	(17)	(18)	(19)
(1) Openness																			
(2) Conscientiousness	.06																		
(3) Neuroticism	.11	.04																	
(4) Agreeableness	.08	.10																	
(5) Extraversion	.14	.00	.14	.03															
(6) Empathy	.09	−.13	−.05	.18	.21 *														
(7) Cooperativeness	.18	.06	−.03	.07	−.06	−.17													
(8) Intelligence	.05	−.19 *	.06	.05	−.04	−.02	.02												
(9) TA photo	.22 *	.20 *	.30 **	.18 *	.29 **	.16	.14	.09											
(10) TA mute video	.45 **	.14	.37 **	.36 **	.30 **	.19 *	.28 **	.20 *	.13										
(11) TA audio video	.27 **	.21 *	.18 *	.30 **	.26 **	.20 *	.14	.19	−.05	.08									
(12) DA photo	.03	.09	.39 **	.10	.20 *	.02	−.02	.06	.54 **	.03	−.02								
(13) DA mute video	.24 **	.08	.28 **	.26 **	.31 **	.09	.07	.17	.16	.65 **	.03	−.04							
(14) DA audio video	.03	.20 *	.07	.08	.28 **	.17	.19 *	.10	.03	−.05	.69 **	−.06	−.07						
(15) OA photo	.10	.16	.28 **	.19 *	.22 *	.04	.15	.10	.57 **	.25 **	−.05	.60 **	.25 **	−.10					
(16) OA mute video	.21 *	.23 *	.05	.10	.28 **	.07	.05	.07	.02	.01	.62 **	−.04	.12	.66 **	.04				
(17) OA audio video	.23 *	.12	.11	.14	.25 **	.04	.03	.13	.14	.46 **	−.01	−.02	.79 **	−.01	.30 **	.23 *			
(18) Total TA	.55 **	.29 **	.50 **	.48 **	.43 **	.31 **	.32 **	.27 **	.49 **	.70 **	.59 **	.28 **	.49 **	.37 **	.39 **	.35 **	.36 **		
(19) Total OA	.27 **	.23 *	.20 *	.22 *	.42 **	.12	.13	.14	.38 **	.37 **	.29 **	.24 **	.59 **	.29 **	.63 **	.58 **	.78 **	.56 **	
(20) Total DA	.21 *	.19 *	.44 **	.35 **	.48 **	.17	.14	.17	.47 **	.41 **	.41 **	.53 **	.57 **	.47 **	.46 **	.43 **	.48 **	.72 **	.70 **

Variables 1 to 8 are single-trait accuracy scores. TA = trait accuracy; DA = distinctive profile accuracy; OA = overall profile accuracy. * *p* < .05, ** *p* < .01.
